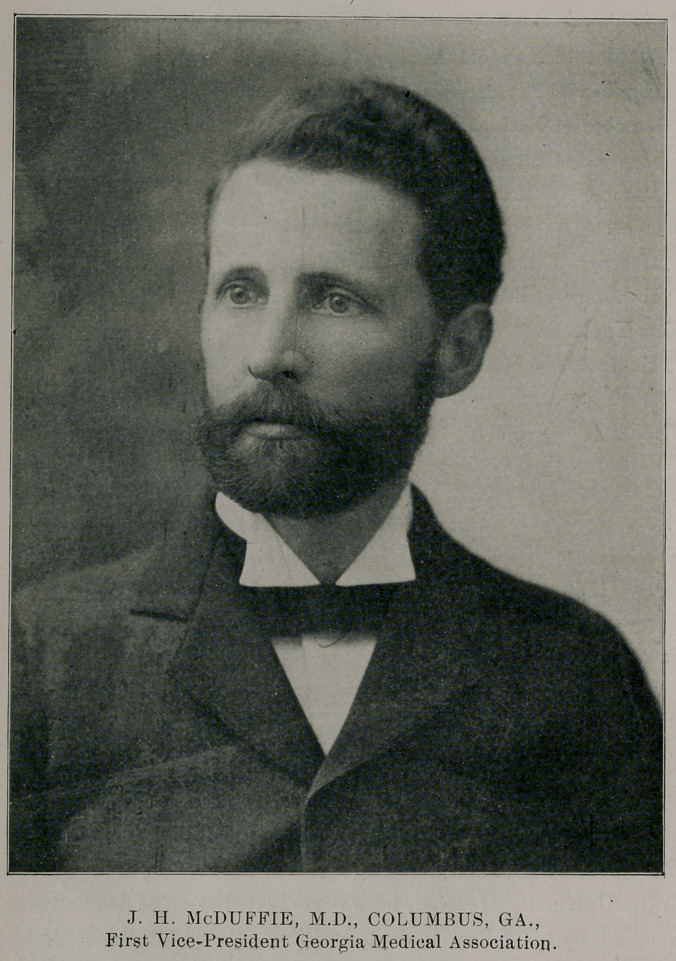# Dr. James H. McDuffie

**Published:** 1903-06

**Authors:** 


### DR. JAMES H. McDUFFIE,
First Vice-President.

THE subject of this sketch was born at Fayetteville, N. C., December 12, 1859, and is a son of James R. and Mary (Johnson) McDuffie. From them he inherits the sturdy old Scotch-Irish blood. His earlier education was received in the high school of his native town. In 1884 an opportunity for carrying out a long cherished desire presented itself, and he began the study of medicine under Dr. James A. Sexton, of Raleigh, N. C. In the fall of 1885 he entered the Medical Department of the University of Maryland, where, after attending lectures and serving as an interne in the University Hospital, he was graduated as an M.D., and was honored by his fellow-students by being elected president of his graduating class. He then returned to North Carolina, and after a successful examination by the State Board, began active practice at Keyser, N. C. Dr. McDuffie spent the winter of 1888 in New

J. H. McDUFFIE, M.D., COLUMBUS, GA.,

First Vice-President Georgia Medical Association.

York city, in postgraduate work, and in March, 1889, removed to Anniston, Alabama, where, after another examination, by the Alabama Board of Censors, he resumed practice. He was shortly afterwards elected a member of the Calhoun County (Ala.) Board of Censors, and also secretary of the County Medical Society—which position he filled until July, 1892, when he removed to Columbus, Ga., where he has since resided. He was most happily married, December 5, 1882, to Miss Sallie H. Page, an accomplished daughter of Louis A. Page, a prominent citizen of Cary, N. C., and seven children—six of whom are now living—have blessed their union. Dr. McDuffie is a member of the American Medical Association and of the Medical Association of Georgia; Vice-President of the Georgia Pasteur Institute and Laboratory in Atlanta; a Knight of Pythias, and an Elder of the First Presbyterian Church of Columbus, Ga.

---

DR. F. W. MCRAE, Dr. L. Amster and Dr. G. H. Noble attended the meeting of the A. M. A. at New Orleans.

---

THE marriage of Miss Frances Talbot, of Eatonton, and Dr. V. O. Hardon occurred on June 2. Congratulations.

---

AT the meeting of the American Academy of Medicine, held in Washington, D. C., May 11 and 12, the following officers were elected for the ensuing year: President, Dr. John B. Roberts, of Philadelphia; Vice-Presidents, Drs. T. B. Davis, of Pittsburg; J. H. McBride, of Pasadena, Cal.; J. P. Searcy, of Tuscaloosa, Ala., and S. A. Knopf, of New York; Secretary, Dr. Charles McIntire, of Easton, Pa.; Assistant-Secretary, Dr. A. R. Craig, of Columbia, Pa.; and Treasurer, Dr. John E. A. Green, of Easton, Pa. The next meeting will be held at Atlantic City, N. J., June 11 to 13, 1904.

## Figures and Tables

**Figure f1:**